# Prevalence of stunting and associated factors among neonates in Shebadino woreda, Sidama region South Ethiopia; a community-based cross-sectional study 2022

**DOI:** 10.1186/s12887-023-04080-4

**Published:** 2023-06-02

**Authors:** Gizu Tola, Andargachew Kassa, Melkamu Getu, Bekem Dibaba, Shambel Neggesse

**Affiliations:** 1 Department of Midwifery, College of Health Science, Mettu University, Mettu, Ethiopia; 2 Department of Midwifery, College of Medicine and Health Science, Hawassa University, Hawassa, Ethiopia; 3 Department of Midwifery, Jimma University, Jimma, Ethiopia

**Keywords:** Stunting, Neonates, South Ethiopia

## Abstract

**Introduction:**

Stunting is a syndrome that begins at conception and leads to severe, irreversible physiological, physical and cognitive damage as an irreversible consequence of nutritional deficiencies and recurrent infections. Although multiple studies have been conducted in Ethiopia to show the magnitude of stunting and factors, all are concentrated on children aged between 6 to 59 months. Therefore, this study was done to determine the prevalence and associated factors of stunting at birth among new-borns.

**Methods:**

A community-based cross-sectional study design was employed on 512 neonates in Shebadino Woreda, Sidama Region South Ethiopia 2022. A multistage sampling technique was employed. The data was collected door-to-door using pretested and structured questionnaires, through face-to-face interviews. The collected data were cleaned manually, coded, entered into Epidata version 4.6, and exported to SPSS version 26 software for analysis. Bi-variable analysis was conducted to assess the association of independent variables with the outcome variable. Variables with a *p*-value < 0.25 in bi-variable logistic regression were further analyzed using multivariable logistic regression. The odds ratio (OR) with 95% CI was used as a measure of association, and variables that had a *p*-value less than 0.05 in the multivariable logistic regression were considered as significantly associated variables.

**Result:**

The prevalence of stunting in this study was 27.5%: 95% CI 22.6 to 31.9. Factors such as residence (AOR = 4.1, 95% CI: 1.49, 11.25), ANC follow up (AOR = 2.66, 95% CI: 1.34, 5.27), started taking Amessa (AOR = 3.48, 95% CI: 1.27, 9.55) and Sex of the neonate (AOR = 2.15, 95% CI: 1.54, 5.23) were significantly associated with stunting at a *p*-value of < 0.05.

**Conclusion:**

About 27% of neonates were stunted, which implies, it require a quick public health measurement. New-born who were live in rural area and those who were started traditional medication (Amessa) were more stunted. Besides this, stunting was prevalently observed among a mother who had no ANC follow-up and male neonates. Thus, the regional health bureau and Shebedino woreda health office should increase awareness creation to bring behavioural change at community level to prevent traditional medication usage, ANC follow-up and giving priority for those who live in rural area.

## Background

Neonatal Stunting is characterized by short length to gestational age,unlike children older than 6 months of age [1, 2]. It is a syndrome that begins at conception and leads to severe, irreversible physiological, physical and cognitive damage as an irreversible consequence of nutritional deficiencies and recurrent infections [3, 4]. At 3 months and 2 years of age, respectively, stunted neonates are four times and two times more likely to be Continues as stunted [5].

Neonatal stunting is caused by a variety of maternal and extra-maternal nutritional and economic problems. Maternal short stature, maternal malnutrition, maternal illness (DM, hypertension, anemia), lack of preventive health care (ANC), male sex, and low birth weight were determinants of growth retardation [1, 2, 6–8]. The long-term effects of stunting range from acute infections to chronic non-communicable diseases such as diabetes, stroke, diarrhoea and, hypertension.

One in three children under five in the world is malnourished. In 2018, around 155 million children under the age of 5 were affected by stunting worldwide. Asia and Africa contribute 56 and 38%, respectively, of the global stunting burden [3]. Stunting affects approximately 144 million children under the age of five, 92% of whom live in low- and middle-income countries and sub-Saharan Africa [4]. Wasting and stunting alone lead to 64.6 million and 54.9 million disability-adjusted life years for children under five, representing 14.8 and 12.6% of all global disability-adjusted life years, respectively [9, 10].

In Ethiopia, the country with the highest burden of malnutrition, 44 and 27% of children under 5 years and under 6 months are affected, respectively [2, 11]. Despite the fact that the country has signed national dietary guidelines and is working to meet the World Health Assembly target of 26.8% stunting by 2025, the annual rate of reduction in stunting is only 2.8%, well below the expected reduction of 6%. Our Country Ethiopia loses about 16.5% of Ethiopia’s Gross Domestic Product (GDP) each year as a result of the long-term negative effects of childhood stunting [12]. This was due to the fact that about half of infant and child mortalities in Ethiopia are associated with stunting and other forms of malnutrition, resulting in an 8% decline in the country’s workforce and affect economic growth [13]. According to the 2019 Ethiopian Mini-Demographic and Health Survey (EDHS), stunting was 17.1% in children under 6 months of age [14]. A recent study conducted in the Wondo Genet Sidama region showed a prevalence of stunting in children under 5 years of age was 50.3% [8].

Most studies on stunting in Ethiopia have focused on children aged 6–59 months, suggesting that the nutritional status of new-borns has been overlooked, but the impact is likely to be significant. There are many initiatives to address malnutrition in children under five, but these strategies need to be supported by data to identify the problem and its causes. In addition, consistent and up-to-date information on early childhood stunting is critical to unlocking the full potential of global policy, such as lowering stunting by 40% by 2025 [15]. Therefore, the purpose of this study is to determine the prevalence and factors associated with stunting among neonates in Shebadino Woreda, Sidama region of southern Ethiopia. This will be helpful for modifying the existing policy, based on the result of the present research, and to deliberate equity-driven policy targeting interventions for the most vulnerable population as early as possible. Additionally, it will serve as a baseline to make comparisons with children aged 6 to 59 months to appreciate the problem among the population.

### Research hypothesis

1. Neonatal stunting is more prevalent in Shebedino woreda
2. Neonates who were delivered from malnourished mother were more likely to be stunted.
3. Being a low income family has a relationship with stunting.

## Methods and materials

### Study settings and design

The study took place from Jun 7/2022 to July 21/2022 in Shebadino woreda, which is located in the Sidama National Regional State, which is approximately 27 km from Hawassa, the state seat, and 310 km from Addis Ababa, Ethiopia’s capital. It is located at an elevation of 1750–3000 m above sea level. According to Shebadino District Health office population profile, there were around 6960 neonates delivered in the woreda last year [16]. The common health problem of the woreda is pneumonia according to studies [17].

#### Population

Study population were neonates with their mothers in selected kebeles of Shebadino woreda, during the actual data collection period. However, neonate’s mothers who were stayed less than 6 months in the woreda were excluded from the study. This was due to some traditional malpractice usage in specific area. Further, new-borns whose mothers suffering from critical illness (postpartum haemorrhage) and Postpartum Psychosis were also excluded. Neonates delivered before 33 completed weeks were also excluded.

### Sample size and sampling procedure

Separate sample size was calculated for each specific objective (to determine the prevalence of Neonatal Hypothermia and to identify the factors associated with Neonatal Hypothermia) using single and double population proportion formulas. The sample size for the first was calculated using the single population proportion formula with the following assumptions: *n* = minimum sample size required for the study, (Z α/2)2 = standard normal distribution with 95% CI and d = a tolerable margin of error (d = 0.05). p = Prevalence of Stunting 30.5% from a previous study conducted in University of Gonder [2]. Then 1.5 for design effects multiplied us, 325.6 × 1.5 = 488. The final sample size derived by adding a non-response rate of 5%, which takes the total sample size to 512.

Multi-stage sampling technique used to select study subjects. There are 30 kebeles in the district, (Twenty-five kebeles from rural, and five kebeles from urban). A simple random sampling technique was employed to select 40% (12) of the total kebeles (the smallest administrative units in a given district in Ethiopia). In selected 12 kebeles, there were 1162 neonates born in two and half months. The calculated sample size was allocated proportionally to the size of the populations in each selected kebeles. Then participants were selected by using a systematic random sampling technique in order of birth registration from HEW, that is, every two birth reports until the required sample size was obtained (K = 2.3, approximately every 2 neonate birth reports was taken). Households of the women who gave birth were identified and reached with the help of the HEW and Health development army of Selected Kebeles.

### Operational definitions

#### Neonatal stunting

Stunted neonates were those with a length-for-gestational-age below the 10th percentile, whereas non-stunted neonates were those with a length-for-gestational age above the 10th percentile.

#### Maternal under nutrition

Mothers whose MUAC measurement < 22 cm [18].

#### Large-for-gestational age

Neonates whose birth weight-for-gestational age > 90th percentile [19].

#### Appropriate-for-gestational age (AGA)

A neonates whose birth weight-to-gestational age fall between 10 and 90th percentile [19].

### Small weight-for-gestational age (SGA)

A neonates whose birth weight-to-gestational age is less than 10th percentile [19].

#### Wealth status

Participants in the first, second, third, fourth, and fifth ranks were classified as richest, richer, middle, poorer, and poorest, respectively, using the Principal Component Analysis [20].

### Data collection tools and procedures

Two MSc for supervision and six Bsc midwives participated in data collection and measurements conducted as soon as possible after birth at the mother’s home. All data collectors and supervisors selected by their previous experience in data collection, and who can able to listen, speak, write, and read Sidama affo and English language. The data collector reached each household, with the help of HEW, and the health development army in each sub kebeles and collects the data after birth. For those mothers who gave birth at health institutions, data taken after discharged to home as soon as possible. A structured, interviewer-administered questionnaire, adapted and modified from the study conducted wondo Genet woreda [8] and University of Gonder [21]. The interview questionnaire was prepared in English, then translated into the local language, Sidamo affo, and then translated back to English by a third person to check for consistency. Data collectors was trained on measuring the length and, the weight of the baby and calculating gestational age and supervised by a team assigned as supervisors.

The weight was measured by using a weighing scale model of RGZ 20 which had a precision to the nearest 50 g [22]. The new-born’s length was measured from the top of their head to the heel of their foot. The measurement was done three times by using infantometer (ITEM CODE: WS025 and SIZE18’X7’). The average length of three times measurements were recorded to the nearest 0·5 cm accuracy. The length and the weight of the new-born was determined by comparing the same-sex references. The INTERGROWTH-21st standard software was used to generate a composite variable, length-for-gestational age whereas, gestational age was estimated from the woman’s report of last menstrual period and early Ultrasound. Maternal Mid Upper Arm Circumference (MUAC) was measured by using fiber tape from the left upper arm at the mid-point between the tip of the shoulder and the tip of the elbow. It was measured twice to ensure its accuracy.

### Data quality assurance

The pretest of the data collection was carried out on 5% of the sample size in Arbegona woredas, on 26 neonates, which have a similar setup to our study area. The purpose of this pretest was to check for the accuracy of responses, language clarity, and appropriateness of the data collection tool, as well as to estimate the time required. Intensive training was given to data collectors for 2 days on information about the research objective, eligible study subjects, data collection tools and procedures, and interview methods. All the collected data were checked for completeness by data collectors and supervisors every day as well as the principal investigator before data entry. Beyond this, the incomplete questionnaires that were missed greater than 10% of the total response were excluded and counted as non-respondents. Weight balance were calibrated’ (the measurement was crosschecked with the reference every week to avoid any false readings due to possible damages during data collection.

### Data processing and analysis

The data was entered into Epidata version 4.6 and then exported to SPSS (Statistical Package for Social Sciences, version 26) for data analysis. Descriptive statistics like frequencies, proportion, and summary statistics (mean and standard deviation) were used to describe the study population in relation to relevant variables and presented in tables, and graphs. Assumptions such as dichotomous, multi-co linearity issue, Chi-square test, and mutually exclusiveness were first checked and then bi-variable analysis was carried out to identify candidate variables (*p* < 0.25) for multivariate analysis. Using Variables found to have a *p*-value < 0.25 in bi-variable analysis were further analyzed using multivariable logistic regression to control the confounder. The odds ratio (OR) with 95% CI was used as a measure of association, and variables that had a *p*-value less than 0.05 in the multivariable logistic regression were considered as significantly associated variables.

Hosmer and Lemeshow test were used to test the goodness of fit which was sig = 0.989 whereas multi-co linearity was checked by variance inflation factors (VIF) which should be < 10. Data normality was checked by using a histogram and Q-Q plot test.

## Result

### Socio-demographic characteristics of respondents

A total of 498 mothers with neonates were included in this study with a 97.3% of response rate. The majority of the participants 334(67%) were in the age group of 20–30 years with a mean of 26 years. 464 (93.2%) of participants were married, whereas 347 (69.7%) were Protestant religious followers. Regarding mothers’ Occupations, 279 (56%) were housewives. About 414 (83%) were from a rural area, whereas 142 (28.5%) respondents had middle wealth index (Table 1).

**Table 1:** Socio-demographic characteristics of neonates mothers in Shebadino wereda, Sidama, Ethiopia 2022 [*n* = 498]

Variables	Frequency	Percent
Mother’s Age		
20	96	19.3
20–30	334	67
30	68	13.7
Marital Status		
Married	464	93.2
Divorced	11	2.2
Single	23	4.6
Religion		
Protestant	347	69.7
Orthodox	112	22.5
Muslim	39	7.8
Occupation		
Housewife	279	56
Governmental employee	17	3.5
Private business	65	13
Farmer	84	16.9
Other (Student)	53	10.6
Husbands Occupation (*n* = 475)		
Farmer	231	48.6
Private business	163	34.3
Governmental employee	68	14.3
Other	13	2,8
Educational status		
Unable to read & write	97	19.5
Read & Write	129	25.9
Elementary school	135	27
High school/preparatory	117	23.5
Above grade12	20	4
Husband’s educational status (*n* = 475)		
Unable to read & write	138	29
Read & Write	69	14.5
Elementary school	102	21.5
Highschool/preparatory	92	19.4
Above grade12	74	15.6
Mother has her own income		
Yes	139	28
No	359	72
Wealth index		
Richest	28	5.6
Richer	67	13.7
Middle	142	28.5
Poorer	104	20.8
Poorest	157	31.5
Residence		
Urban area	84	16.7
Rural area	414	83

### Obstetric characteristics of the mothers

Four hundred two (80.7%) of the mothers had visited health facilities for antenatal care (ANC) during the recent pregnancy at least one time. 54 (10.8%) of the mothers reported that they had obstetric problems during their most recent pregnancy, with Hypertension being the most commonly reported problem (42.6%). The majority, 368 (73.4%), of the mothers were delivered at a health facility. 205 (73%) were delivered at > = 37 completed weeks. About 96.4% of the neonates were delivered single (Table 2).

**Table 2:** Obstetric characteristics of the neonates mothers in Shebadino woreda, Sidama region, Ethiopia 2022 (*n* = 498)

Variable	Category	Frequency	Percent
ANC follow up during the last pregnancy	Yes	402	80.7
	No	96	10.3
Number of ANC visits (*n* = 402)	< 4	286	71
	> = 4	116	29
MUAC	Normal	346	69.5
	Chronic malnutrition	152	30.5
Obstetric problem during the last pregnancy/labor (*n* = 416)	Yes	54	10.8
	No	444	89
Type of the obstetrical problem (*n* = 54)	Bleeding	8	2.9
	Hypertension	23	42.6
	PROM	17	31.5
	DM	6	11
Place of birth	Health Facility	368	73.4
	Home	130	26
**Short inter-birth interval**	Yes	68	13.7
	No	430	86.3
Gravidity	Multigravida	370	74.3
	Primigravida	128	25.7
Parity	1–3	418	84
	4–6	65	13
	> 6	15	3
Gestational age in weeks	< 37 weeks	76	15.3
	> = 37 weeks	422	84.7
Number of the child delivered	Single	477	96
	Twin	21	4

### Neonatal characteristics and maternal feeding factors

Two hundred fifty nine (52%) of the Neonates were Females. Of the total participants, 277 (55.5%) of the neonate were greater than 7 days of age with a mean age of 9.01. 102 (20.5%) of the neonates manifested different symptoms like a decrease in neonatal movement and red umbilicus which accounted for 11% and 7.6%, respectively. 241 (48.4%) of the neonates were given the traditional medication “Amessa” after delivery. 453(91%) of the mothers initiated breast feeding within the first 1 h, whereas 393(79%) neonates had optimal breast feeding (Table 3).

**Table 3:** Behavioral and neonatal factors among neonates in Shebadino woreda, Sidama, South Ethiopia 2022 (*n* = 498)

Variables	Category	Frequency	Percent
Baby breastfed within 1 h after delivery	Yes	453	91
	No	45	9
Optimal Breast feeding	Optimal	393	79
	Suboptimal	119	21
Traditional practice applied to baby (Amessa given)	Yes	241	48.4
	No	257	51.6
Baby took food by mouth	Yes	38	7.6
	No	460	92.4
Types of food taken Orally (*n* = 38)	Water	14	36.8
	Milk	18	47.4
	Butter	6	15.8
Age of the neonate in days	0–7 days	221	44.4
	8–28 days	277	55.6
Babies presented with clinical S/S	Yes	102	20.5
	No	396	79.5
Clinical manifestation raised (*n* = 102)	Red umbilicus or pus	38	7.6
	Fast breathing	40	8
	Not moving well	55	11
	Fever	19	3.8
	Vomiting	49	9.8
Sex of the neonate	Male	239	48
	Female	259	52

### Anthropometric measurement

The mean weight of the neonate was 3445.59 g (± SD 327.23 g); where the median neonates length was 51 cm at 2nd percentile. The mean (± SD) maternal MUAC was 23.28 cm (± 2.4 cm) whereas the median gestational age was 39 weeks (IQR = 2 weeks). About 152 (30.5%) of mothers were chronically malnourished.

### Prevalence of neonatal stunting

The prevalence of stunting in this study was (27.5%: 95% CI 22.6 to 31.9).

### Factors associated with neonatal stunting

Factors that were found to be significantly associated with neonatal Stunting in the bivariable analysis were Family residence, ANC follow up, Separated Human and animals house, Babies with other sign and symptoms, Started taking Amessa, hx of pregnancy problem, Nutritional status of the mother, started breastfeeding within 1 h, Sex of the neonate and wealth index. Factors such as residence, ANC follow-up, Residence, started taking Amessa and Sex of the neonate were significantly associated with stunting at a *p*-value of < 0.05 (Table 4).

**Table 4:** Bi-variable and multivariable logistic regression model with cross tabulation for factors associated with neonatal stunting among neonates in Shebadino woreda, Sidama Region, South Ethiopia 2022 (*n* = 498)

**Variable**	**Stunted (137)**	**Non-Stunted (361)**	**COR (95% CI)**	**AOR (95% CI)**
**Residence**				
Rural	94 (22.7%)	320 (77.3%)	4.7(3.67–8.59)*	4.1 (1.49–11.25)***
Urban	43 (51.2%)	41 (48.8%)	1	1
**ANC follow up**				
Yes	83 (20.6%)	319 (79.4%)	6.84 (4.6–10.12)*	2.66 (1.34–5.27)**
No	54 (56.25%)	42 (43.75%)	1	1
**Separated Human & Animals House**				
Yes	48 (14%)	296 (86%)	3.54 (2.38–5.26)*	2.49 (1.79–3.22)
No	89 (46.4%)	65 (53.6%)	1	1
**Babies with other S/S**				
Yes	69 (67.6%)	33 (32.4%)	2.24 (1.44–3.47)*	1.61 (0.52–4.93)
No	68 (17.2%)	328 (82.8%)	1	1
**Started taking Aamessa**				
Yes	74 (26.1%)	167 (69.3%)	3.04 (1.55–5.97)*	3.48 (1.27–9.55)***
No	63 (24.5%)	194 (75.5%)	1	1
**Hx of px problem**				
Yes	31 (57.4%)	23 (42.6%)	2.2 (1.56–3.1)*	2.10 (0.40–11.1)
No	106 (23.9%)	338 (76.1%)	1	1
**Nutritional status of the mother**				
Normal	56 (16.2%)	290 (83.8%)	3.36 (1.74–6.49)*	0.51 (0.14–1.84)
Chronic malnutrition	81 (53.3%)	71 (46.7%)	1	1
**Started BF within 1 h**				
Yes	33 (76.7%)	10 (23.3%)	8.7 (3.07–24)*	2.86 (0.76–10.72)
No	285 (52.8%)	255 (47.2%)	1	1
**Sex of newborn**				
Male	74 (30.9%)	165 (69.1%)	4.38 (1.84–7.56)*	2.15 (1.54–5.23)**
Female	63 (24.3%)	196 (75.7%)	1	1
**Wealth index**				
Richest	8 (28.6%)	20 (71.4%)	1	1
Richer	9 (13%)	58 (87%)	1.36 (0.85–2.84)	0.55 (0.34–1.97)
Middle	42 (30%)	100 (70%)	1.02 (0.64–1.51)	0.47 (0.25–0.84)
Poorer	32 (37%)	72 (63%)	0.87 (0.57–2.44)	0.81 (0.73–1.12)
Poorest	46 (30%)	108 (70%)	1.30 (0.95–3.52)	0.89 (0.74–2.15)

*Hx* History, *px* Pregnancy, *S/S* Sign, and Symptom, *PO* Per Os/oral

Key. P* Candidate for multivariable at *p*<0.25, P** Significant at *P*<0.05; P*** Significant at *P*<0.01

## Discussion

The prevalence of stunting in this study was (27.5%: 95% CI 22.6 to 31.9), which shows a much more than the 2025 SDG target and still high public health problem [23]. But it was lower than the whole Ethiopian profile 37% [14]. This study finding is in line with the studies conducted in University of Gonder 30.5% [2]. This similarity may be due to the country’s demographic similarity and study subjects (both studies conducted on neonates). However, This study was lower than a study done in Guatemala 38% [24]. This may be countries’ Socio Economic and cultural difference related to our study area. On the contrary, it is higher than study conducted in Indonesia [25]. The possible justification for this difference may be due to the Economic discrepancy between two nations. This study was also lower than study conducted in Wondo Genet Sidama region 50.3% [8]. This variation may be due to time gap between two studies. Additionally this study was included only neonates whereas the first study includes all under-five children.

According to this study, Neonates who were delivered from a mother who had no history of ANC follow up were 2.7 times more likely to develop stunting with related to their counterparts. This may be due to lack of a birth preparedness and creating favorable environment for traditional child feeding mal- practices that might affect child nutritional status [26]. This study was supported by study conducted in Afar region and 2016 Ethiopian Demographic and Heath Survey (EDHS) data [27, 28].

According to this study, male neonates were 2 times more likely to be stunted than those of female. This study finding is congruent with a study conducted in University of Gonder, Central region of Mozambique and Guatemala [2, 24, 29]. This may be due to females express multiple placental genes and protein changes that result in a milder decrease in growth without actual growth restriction [30]. Additionally, other than the placental growth, male fetuses are liable for adverse events than female associated with the rapid body and brain growth [31].

Neonates from rural residence had a four times more likely to develop stunting than their counterparts. This finding was opposed by study conducted in Zambia, and Data from the Demographic and Health Surveys for 11 countries [32, 33]. This may be due to our study sample, which was done on a single area and nation with cultural and economic discrepancies.

The other significant factor with stunting is neonatal traditional medication (Amessa) feeding. Those neonates who had taken amessa had 3.5 times more likely to develop stunting. This may be due to intervention with exclusive breast-feeding and neonate’s lack of sufficient food. It is new factors added by this study and we can’t able to compare with other previous studies.

Our study was used length-for-gestational age to determine stunting at birth. On the contrary previous studies used just only the length measurement regardless of the gestational age. But our study didn’t assess pre-pregnancy weight, which is the major contributing factor of maternal nutrition. Our study was done in one season, and considerations such as seasonal variations were not taken into account. Additionally, our study shares the limitation of the cross-sectional study. The other limitation of our finding was recall bias. To decrease this possibility proper definition and articulation of the research questions, and administering the interview properly and consistently was done.

## Conclusion

The prevalence of neonatal stunting in our study area was high, 27%, which implies, it require a quick public health measurement. New-born who were live in rural area, those who were started traditional medication (Amessa), a mother who had no ANC follow-up and male neonates were the factors that had a significantly associated with neonatal Stunting. Thus, the regional health bureau and Shebedino woreda health office should increase awareness creation to bring behavioural change at community level to prevent traditional medication usage, ANC follow-up and giving priority for those who live in rural area. NGO working in this area should alert and run for solutions to prevent stunting in the woreda based on our findings and address the gaps. Health institutions should advice the family on nutrition before discharge to home and Health extension community based neonatal care is highly encouraged post -natal.

## What is known about this topic?

- Stunting is one of the major public health problems in Sub Saharan Africa including Ethiopia
- Prevalence was determined for under five children and many initiatives to address malnutrition was done
- Some socio demographic characteristics was related with stunting

## What this study adds?

- The prevalence of stunting among neonates in shebedino woreda Sidama region south Ethiopia was 27.5%
- This study was added factors such as Traditional medication (Amessa) usage which was significantly associated with stunting
- Our study was done on Neonate unlike that of previous study (6–59 months).

## Data Availability

The dataset used/or analyzed during the current study are not publicly available. Because we did not have consent from all participants to publish raw data, but are available from the corresponding author on reasonable request.

## Abbreviations

AOR: Adjusted odd ratio
CI: Confidence interval
CBNC: Community based neonatal care
CFR: Case fatality rate
COR: Crude odd ratio
EDHS: Ethiopia demographic health survey
EMDHS: Ethiopia Mini demographic health survey
ENC: Essential newborn care
HEW: Health extension worker
IRB: Institutional review board
MDG: Millennium Development goals
WHO: World Health organization
